# Deciphering the Role of Adrenergic Receptors in Alzheimer’s Disease: Paving the Way for Innovative Therapies

**DOI:** 10.3390/biom15010128

**Published:** 2025-01-15

**Authors:** Androulla N. Miliotou, Andria Kotsoni, Lefteris C. Zacharia

**Affiliations:** 1 Department of Health Sciences, School of Life and Health Sciences, University of Nicosia, 46 Makedonitissas Avenue, 2417 Nicosia, Cyprus; miliotou.a@unic.ac.cy (A.N.M.);; 2 Bioactive Molecules Research Center, School of Life and Health Sciences, University of Nicosia, 46 Makedonitissas Avenue, 2417 Nicosia, Cyprus

**Keywords:** adrenergic receptors, Alzheimer’s disease, neurodegeneration, neuroinflammation, tauopathies, beta amyloid, adrenergic agonists, adrenergic antagonists

## Abstract

Neurodegenerative diseases are currently among the most devastating diseases with no effective disease-modifying drugs in the market, with Alzheimer’s disease (AD) being the most prevalent. AD is a complex multifactorial neurodegenerative disorder characterized by progressive and severe cognitive impairment and memory loss. It is the most common cause of progressive memory loss (dementia) in the elderly, and to date, there is no effective treatment to cure or slow disease progression substantially. The role of adrenergic receptors in the pathogenesis of Alzheimer’s disease and other tauopathies is poorly understood or investigated. Recently, some studies indicated a potential benefit of drugs acting on the adrenergic receptors for AD and dementias, although due to the heterogeneity of the drug classes used, the results on the whole remain inconclusive. The scope of this review article is to comprehensively review the literature on the possible role of adrenergic receptors in neurodegenerative diseases, stemming from the use of agonists and antagonists including antihypertensive and asthma drugs acting on the adrenergic receptors, but also from animal models and in vitro models where these receptors have been studied. Ultimately, we hope to obtain a better understanding of the role of these receptors, identify the gaps in knowledge, and explore the possibility of repurposing such drugs for AD, given their long history of use and safety.

## 1. Introduction

The most widespread and common type of dementia is Alzheimer’s disease (AD), which poses a significant challenge to global health and inflicts devastating consequences on patients, caregivers, and health systems. It is a progressive illness that leads to cognitive impairment, loss of memory, and inability to perform even simple daily living tasks in the end stages [1,2]. AD affects millions, and that number is expected to increase substantially owing to the aging population [3]. Despite the heavy research on AD, no disease-modifying treatments are available that could alter the course of AD. Current treatment options are largely symptomatic in nature and result in slight and short-lived benefits in cognitive function without modifying the mechanisms responsible for neurodegeneration [4].

AD is a complex disorder that is associated with multiple pathological features such as amyloid-beta (Aβ) accumulation, hyperphosphorylation of tau protein, neuroinflammation, loss of synapse connectivity and cardio-vascular abnormalities [5,6,7]. All of these processes are interrelated, which eventually result in a loss of neurons. Recently, research has started focusing on the noradrenergic system, more specifically on adrenergic receptors (ARs), and their role in the development of Alzheimer’s disease as a significant contributing factor [8,9,10,11,12,13,14]. Adrenergic receptors are GPCRs whose primary function is to respond to norepinephrine (NE) or epinephrine in the brain where such cognitive, vasculature and inflammatory activities are regulated. Such dysregulation, with subsequent loss of the non adrenergic system and innervation, is caused by the early degeneration of the locus coeruleus (LC), which is one of the few regions that produces NE and is affected in AD [11,15,16,17].

Adrenergic receptors, divided into α and β, each have a different function and signaling pathways. Such receptors are present in most parts of the Central Nervous System (CNS) and are important in the control of synaptic plasticity, neuroinflammation, neurovascular coupling and various metabolic activities. Degeneration of the LC also occurs in AD, leading to a dramatic hypofunction of NE signaling, whose neural effects are likely to potentiate the toxicity of Aβ, promote the accumulation of tau, and damage neurons. What is more, adrenergic receptors can both protect from AD and increase its risk, which is dependent on the subtype of the receptor, its activated form, and the place where it is situated [18,19]. This presents opportunities for drug design but also points to problems associated with using them effectively in practice.

Given the extensive expression of adrenergic receptors and their multifunctional roles, they are considered promising targets for drug development for AD. There are many drugs targeting adrenergic receptors that are currently being used in clinical practice for hypertension, bronchial asthma, and some psychiatric disorders. This is advantageous since it allows for the repurposing of such drugs for the treatment of AD without extensive clinical trials. However, whether they can be beneficial is still under debate, as in some cases they seem to be beneficial, but not always. Thus, potential modulation of these receptors should be carried out in a very specific manner based on receptor subtypes, brain regions, and disease stage.

**Figure 1:**
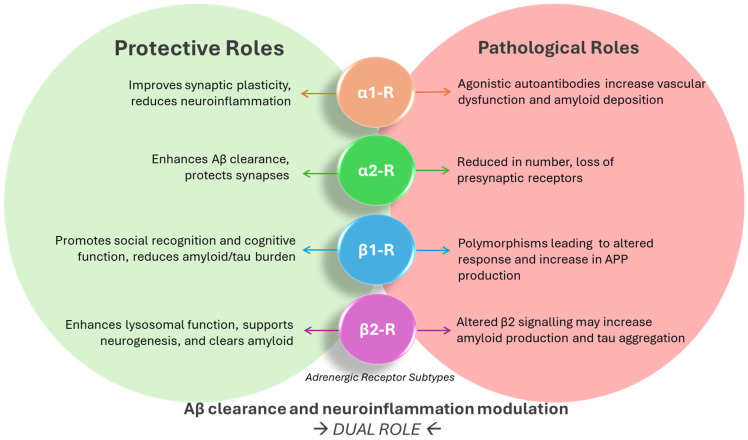
A visual summary of the dual roles of adrenergic receptors in Alzheimer’s disease.

However, whether activation or blockade of the adrenergic receptors is beneficial is not clear. This review evaluates the evidence regarding the role of adrenergic receptors in AD in a consolidated and comprehensive manner, drawing on insights from preclinical models, genetic studies, and clinical trials. An overview of the role of the dual role of adrenergic receptors is found in Figure 1. By synthesizing these findings, the review aims to elucidate the critical importance of adrenergic receptor modulation in the pathophysiology of AD and its potential as a therapeutic target. In particular, it aims to delineate whether evidence points to whether activation or inhibition of adrenergic receptors is likely protective, and the possibility that such drugs can be designed or existing ones repurposed. In doing so, it will explore how adrenergic signaling influences key pathological processes, including Aβ accumulation, tau hyperphosphorylation, neuroinflammation, and synaptic dysfunction, while also addressing the dual protective and pathological roles of these receptors. The overarching goal is to pave the way for innovative, mechanism-driven therapies that can harness the therapeutic potential of adrenergic receptor modulation, while mitigating its detrimental effects, to provide transformative solutions for patients suffering from AD and related neurodegenerative disorders.

### 1.1. α-Adrenergic Receptors

Among the adrenergic receptor subtypes, α-ARs stand out for their nuanced roles in neurodegeneration, particularly in AD and related tauopathies. This is a group of G-protein-coupled receptors which are classified into α1 and α2 adrenergic receptors. They are naturally activated by norepinephrine (NE) and are important for synaptic plasticity, neurovascular activities, and inflammation [20]. Due to their presence in dense innervation and modulating effect on Aβ processing and tau pathology, they are positioned at the center of neurodegeneration associated with Alzheimer’s disease pathology.

The α-ARs therapeutic efficacy is complicated by their dualism. α1-ARs have the most pronounced effect and have shown to be neuroprotective in preclinical studies, where activation has been associated with enhancement of synaptic function, reduced neuroinflammation, and enhanced cognitive outcomes [9]. Likewise, selective α2-AR agonists decrease the amyloidogenic processes while increasing the neurogenic processes, both very useful in terms of treatment. Still, those receptors pose some issues. In some patients with AD, there are circulating agonistic α1-AR autoantibodies that cause chronic overstimulation of those receptors, which leads to neurotoxic and vasculotoxic effects [21]. With respect to α2-ARs, however, the side effects may include the promotion of amyloid production and pathological amyloid precursor protein (APP) activation upon receptor stimulation [22].

In recent years, studies on genetic, molecular, and pharmacological aspects of α-ARs advances have greatly enhanced understanding of their roles in the pathophysiology of AD. This section examines α-ARs in AD and discusses their positive and negative effects, addressing how they can mediate the control of dementia.

### 1.2. Positive Roles of α-Adrenergic Receptors in Alzheimer’s Disease and Other Neurodegenerative Disorders

#### 1.2.1. α1-Adrenergic Receptors

The α1 adrenergic receptors (α1-ARs) have emerged as a focal point of interest in the context of AD and other neurodegenerative disorders due to their potential to influence key mechanisms underlying neuronal function and pathology. The therapeutic potential of these receptors is underscored by studies demonstrating both the protective effects of their activation and the benefits of selective inhibition. These seemingly contradictory findings emphasize the complexity of α1-AR signaling and its role in neurodegeneration.

One promising development in this area is the discovery of positive allosteric modulators (PAMs) for α1A-ARs. Notably, Compound 3 (Cmpd-3), a PAM for α1A-ARs, has been shown to rescue LTP defects and normalize amyloid β (Aβ)-40 and –42 levels in AD mouse models. These effects were achieved without impacting blood pressure, which is a common concern in adrenergic receptor-targeted therapies. Furthermore, oral administration of Cmpd-3 at doses ranging from 3 to 9 mg/kg once daily for three months led to significant cognitive improvements that outperformed donepezil, which is the standard acetyl cholinesterase inhibitor treatment for AD. These findings highlight the potential for PAM like Cmpd-3 as a disease-modifying agent with robust therapeutic efficacy [23]. Furthermore, mice with constitutively active α_1A_AR showed enhancements in learning and memory compared to α_1A_AR knockout. WT mice treated with the α_1A_AR-selective agonist cirazoline also showed enhanced cognitive functions, suggesting that long-term α_1A_AR stimulation improves synaptic plasticity, cognitive function, mood, and longevity [24]. Further supporting the neuroprotective role of α1-ARs, avenanthramide-C, a compound derived from oats, has demonstrated the ability to reverse memory impairments in AD models Th2529 and 5XFAD. This compound improves recognition and spatial memory and reduces neuroinflammation, effects that are mediated by its interaction with α1A-ARs. The beneficial effects of avenanthramide-C were abolished by prazosin, a specific α1A-AR inhibitor, confirming the critical role of these receptors in protecting [25]. Interestingly, studies on α1B-AR knockout mice have highlighted the critical role of this receptor subtype in cognitive processes. Mice deficient in α1B-ARs exhibited significant impairments in memory consolidation and exploratory behavior, indicating that α1B-ARs may support cognitive function when activated [26]. These findings suggest that modulation of specific α1-AR subtypes could yield differential therapeutic outcomes depending on the context of receptor activity and the disease state.

Despite these data, pharmacological inhibition of α1-ARs has also revealed neuroprotective effects in certain contexts. Doxazosin, an α1-adrenergic blocker, has been shown to protect hippocampal slices from amyloid-β toxicity by preventing glycogen synthase kinase-3β (GSK-3β) activation and tau hyperphosphorylation [27]. Similarly, prazosin reduced Aβ generation, induced anti-inflammatory responses, and prevented memory deficits in transgenic APP23 mice despite not affecting amyloid plaque load. These findings underscore the anti-inflammatory and neuroprotective benefits of α1-AR antagonists in AD [28]. Another α1-AR antagonist, terazosin, has demonstrated a novel mechanism for mitigating neurodegenerative pathology. By increasing ATP levels and enhancing autophagy, terazosin reduced pathological protein aggregates in AD and other neurodegenerative disease models, highlighting its potential as a treatment strategy [29]. Terazosin was also shown to reduce amyloid plaque burden, tau hyperphosphorylation, and glial activation. Behavioral deficits in AD models were also significantly improved with terazosin treatment. These findings suggest that selective inhibition of α1-ARs may counteract pathological processes underlying AD, offering a potential therapeutic pathway [30].

#### 1.2.2. α2-Adrenergic Receptors

The α2 adrenergic receptors (α2-ARs) have also been implicated in neuroprotection and cognitive enhancement in AD and other neurodegenerative disorders with mixed results, as both activating and blockading have been shown to be neuroprotective, with most studies favoring blockade rather than activation for protection. Genetic studies have provided further evidence for the protective role of α2-ARs. A deletion variant of the α2b-adrenergic receptor has been associated with enhanced memory formation and a reduced risk of developing AD in 311 Greek subjects. This variant was more prevalent in control subjects compared to individuals with AD or mild cognitive impairment, suggesting its potential as a protective genetic factor [31].

Pharmacological modulation of α2C-ARs (α2 subtype C) has shown promise. ORM-10921, a selective α2C-AR antagonist, improved cognitive function (measured by water maze) and alleviated symptoms in CNS disorders, including AD. Its high selectivity for the α2C-AR subtype supports its potential as a targeted therapeutic for neurodegenerative diseases [32]. In addition to these pharmacological findings, the α2-adrenoceptor antagonist dexefaroxan has been found to enhance neuron survival, offering a novel approach for preserving cognitive function in neurodegenerative conditions. Dexefaroxan also reduced cholinergic degeneration and improved memory performance in rodent models, further supporting its therapeutic potential [33,34,35]. Another α2-adrenoblocker, mesedin, has demonstrated multiple neuroprotective effects in vivo, including anti-amyloidogenic action, increasing choline acetyltransferase levels, enhancing Aβ degradation, and reducing neuroinflammation. These findings highlight the multifaceted role of α2-AR modulation in mitigating AD pathology [36].

However, the α2A adrenergic receptor has also been implicated in the enhancement of Aβ generation, linking noradrenergic dysregulation to AD progression. Activation of α2A-ARs disrupts the interaction between APP and sorting-related receptors with A repeat (SorLA), a critical regulator of APP trafficking. This disruption facilitates the redistribution of APP to endosomes, where it is cleaved by β-secretase, leading to increased Aβ production. Blockade of the α2AR with Idazoxan reduced AD-related pathology and ameliorated cognitive deficits in an AD transgenic mode [37].

Agonists such as brimonidine and clonidine have demonstrated substantial neuroprotective effects, particularly by reducing retinal ganglion cell apoptosis. This effect is achieved via reduction in Aβ and APP processing, emphasizing the therapeutic relevance of α2-ARs in conditions characterized by amyloid pathology [38].

### 1.3. Negative Roles of α-Adrenergic Receptors in Alzheimer’s Disease and Other Neurodegenerative Disorders

#### 1.3.1. α1-Adrenergic Receptors

Despite the above evidence, the α1 adrenergic receptors (α1-ARs) have also been associated with several adverse outcomes in AD and other neurodegenerative disorders, underscoring their complex role in disease pathology. Research indicates that their persistent activation and the presence of autoantibodies targeting these receptors contribute significantly to vascular and neuronal dysfunctions.

Agonistic autoantibodies (agAABs) against α1-ARs have been identified in approximately 50% of AD patients. These autoantibodies mimic natural agonists, binding persistently to the receptors and causing prolonged activation. This aberrant signaling leads to non-physiological intracellular calcium elevations, triggering macrovascular and microvascular impairments. In animal models, the presence of these autoantibodies has been linked to significant reductions in blood flow and vessel density, impairing cerebral perfusion and contributing to neurodegeneration. Immunoadsorption has emerged as a potential strategy to mitigate these effects. In a study involving AD patients, the removal of α1-AR autoantibodies through immunoadsorption stabilized cognitive function over a follow-up period of 12–18 months, highlighting the therapeutic potential of this approach [39,40]. Further investigations revealed that 59% of patients with mild to moderate AD and vascular dementia harbor autoantibodies targeting both α1- and β2-adrenergic receptors. These autoantibodies preferentially bind to the extracellular loop of α1-ARs, mobilizing intracellular calcium and exacerbating vascular lesions. Such processes are believed to facilitate amyloid plaque formation and vascular lesions [21].

In addition, the detrimental effects of α1-AR activation extend to cerebral vasculature. Neuroimaging studies on rats demonstrated significant reductions in relative cerebral blood volume (rCBV) within the cerebrum, cortex, and hippocampus due to α1-AR autoantibody activity. These findings correlate with impaired neurovascular function, a hallmark of AD pathology [41]. The IMAD pilot trial (efficacy of immunoadsorption for treatment of Alzheimer’s disease and agonistic autoantibodies against α1-AR) investigates the potential of immunoadsorption to remove α1-AR autoantibodies and mitigate their harmful effects as suggested by previously mentioned studies on autoantibodies [42].

Furthermore, Aβ peptides have been found to directly activate α1-ARs, contributing to vascular dysfunction. This interaction induces a positive chronotropic effect in cardiac assays and triggers intracellular calcium release in vascular smooth muscle cells. Such effects are attenuated by α1-AR blockers, including prazosin, emphasizing the receptor’s involvement in Aβ-induced vascular pathology [43]. This highlights the detrimental role of α1-AR activation in AD-associated cardiovascular and cerebrovascular abnormalities.

The pathological effects of α1-ARs are not limited to vascular changes but extend to behavioral outcomes. Aggressive behavior in AD patients has been associated with upregulation of α1-ARs. Postmortem studies revealed that increased α1 receptors were correlated with aggressiveness in AD patients, implicating heightened α1-AR activity in behavioral dysregulation [44].

In conclusion, α1-ARs play a multifaceted role in AD and other neurodegenerative disorders, with their pathological activation contributing to vascular dysfunction, amyloid pathology, neuroinflammation, and behavioral changes. The presence of agonistic autoantibodies further exacerbates these effects, while receptor inhibition or immunoadsorption offers potential therapeutic benefits. Continued research into α1-AR modulation and the development of targeted interventions are crucial for addressing the complex pathophysiology of AD and improving patient outcomes.

#### 1.3.2. α2-Adrenergic Receptors

The negative roles of α2AR in neurodegenerative diseases are much less implicated and not widely supported by the literature. In AD brains, a significant reduction in α2-AR density has been observed in the prefrontal cortex, with decreases of approximately 50% compared to age-matched controls. This reduction is attributed to a loss of presynaptic receptors on noradrenergic synapses rather than changes in ligand affinity, highlighting the degeneration of neurons. These findings suggest that α2-AR deficits may contribute to the cognitive and behavioral impairments associated with AD [45,46].

### 1.4. β-Adrenergic Receptors

β-ARs represent a distinct and significant component of the adrenergic receptor family, with notable roles in AD pathophysiology. β-ARs are primarily associated with their ability to regulate intracellular signaling pathways that influence neuroinflammation, synaptic plasticity, and energy metabolism. Comprised of three subtypes—β1, β2, and β3—these G-protein-coupled receptors are activated by NE and epinephrine and are broadly distributed in the brain. They interact with cAMP-mediated pathways, making them key modulators of cellular functions that are directly implicated in AD pathology [47].

This section examines the multifaceted roles of β-ARs in AD, delineating their contributions to both disease progression and potential therapeutic interventions. By exploring the intricate mechanisms by which β-ARs influence AD pathology, this discussion aims to clarify their distinct functions relative to α-adrenergic receptors and highlight their potential as targets for precision medicine in neurodegenerative disorders.

### 1.5. Positive Roles of β-Adrenergic Receptors in Alzheimer’s Disease and Other Neurodegenerative Disorders

#### 1.5.1. β1 Adrenergic Receptors

The β1-ARs have emerged as a promising therapeutic target in AD and other neurodegenerative disorders due to their role in modulating cognitive functions, including memory and social learning. Importantly all studies indicate that the protective effects are mediated by activating the β1 receptor. One critical area of β1-AR activity is in the medial amygdala, where these receptors facilitate the learning and processing of social cues. Dysfunction in social recognition has been effectively addressed in preclinical studies through selective activation of β1-ARs. Xamoterol, a selective partial β1-AR agonist, was shown to rescue social recognition deficits in an AD mouse model (APP) by activating the protein kinase A (PKA)/phosphorylated cAMP-response element-binding protein (phospho-CREB) signaling cascade. This pathway is essential for synaptic plasticity and memory formation, highlighting the potential of β1-AR modulation to restore cognitive abilities specifically related to social interaction in AD [48]. Chronic administration of xamoterol has demonstrated significant reductions in key pathological features of AD, including Aβ and tau pathology. In the 5XFAD mouse model, a widely used AD model characterized by robust Aβ and tau accumulation, xamoterol treatment reduced neuroinflammation markers. It also lowered mRNA expression levels of inflammatory mediators, indicating its potential to modulate both systemic and localized neuroinflammatory processes. The impact of β1-AR activation on AD pathology is further underscored by xamoterol’s ability to attenuate Aβ plaque deposition and tau hyperphosphorylation in vivo [49]. Additionally, chronic nebivolol treatment (a selective β1 adrenergic receptor antagonist) on Tg2576 mice with established amyloid neuropathology and cognitive impairments significantly reduced brain amyloid content but failed to improve cognitive function [50]. These outcomes suggest that β1-AR modulation not only protects against neuronal loss and cognitive decline but also addresses the underlying pathological mechanisms driving AD progression. The therapeutic potential of β1-ARs has also been studied with another selective partial agonist, STD-101-D1. This compound demonstrates selective partial agonistic activity on G-protein signaling with an EC50 value in the low nanomolar range, indicating high efficacy and potency. Functionally selective agonists like STD-101-D1 allow for targeted modulation of β1-AR activity, minimizing side effects associated with full receptor activation. STD-101-D1 has shown dual benefits in neuroprotection and inflammation reduction, both of which are critical in addressing AD pathology. In vitro and in vivo studies have demonstrated that this compound effectively inhibits the tumor necrosis factor-α (TNF-α) response triggered by lipopolysaccharide (LPS), a known inducer of neuroinflammation. Neuroinflammation, a significant contributor to synaptic dysfunction and neuronal loss in AD, is thus effectively mitigated by STD-101-D1, making it a strong candidate for further therapeutic development [51]. The ability of β1-AR-targeted therapies to modulate neuroinflammatory pathways positions these receptors as key targets not only for AD but also for other neurodegenerative and neuroinflammatory disorders. The neuroprotective effects of β1-AR modulation are closely tied to their influence on intracellular signaling cascades. Activation of β1-ARs leads to increased cAMP production, which activates PKA and downstream signaling molecules such as CREB. This cascade promotes the transcription of neuroprotective genes, including those involved in synaptic repair and neuronal survival. Furthermore, β1-ARs appear to regulate neuroinflammation through inhibition of pro-inflammatory cytokines like TNF-α, providing a dual mechanism of action that addresses both neuronal and immune components of neurodegeneration.

#### 1.5.2. β2-Adrenergic Receptors

The β2-ARs have emerged as crucial modulators in the context of AD and other neurodegenerative conditions. By influencing cellular, immune, and synaptic processes, β2-AR activation offers a multifaceted approach to mitigating AD pathology and preserving cognitive function. Their ability to influence cellular clearance processes, immune regulation, and synaptic function positions them as promising therapeutic targets. Central to this is their role in restoring lysosomal function and autophagy, both of which are crucial in mitigating AD pathology. In particular, β2-AR agonists such as isoproterenol have been shown to reacidify lysosomes in presenilin-1 (PSEN1) KO fibroblasts from PEN1 familial AD patients, which restores lysosomal proteolysis, calcium homeostasis and normal autophagy flux [52]. Building on these cellular benefits, β2-ARs also play a significant role in modulating the brain’s immune response. Microglia, the brain’s immune cells, are highly responsive to β2-AR signaling, which protects them from Aβ-induced inflammation. This anti-inflammatory effect has been observed in both pharmacological treatments using β-adrenergic agonists and lifestyle interventions like environmental enrichment. Mice exposed to these interventions showed reduced microglial activation and a decline in pro-inflammatory cytokine levels, highlighting the ability of β2-AR signaling to mitigate harmful neuroinflammatory responses that exacerbate AD pathology [53].

Activation of β2-ARs significantly contributes to preserving synaptic health by preventing LTP inhibition by Aβ protein. Through the cAMP/PKA signaling pathway, activation of β2-ARs prevents Aβ oligomer-induced inhibition of LTP, a key mechanism underlying learning and memory. This synaptic protection underscores the potential of β2-AR-targeted therapies in maintaining cognitive function and lessening the effects of Aβ accumulation during aging [54]. β2-ARs are particularly significant in the context of AD. These receptors regulate lysosomal function and autophagy, which are essential for the clearance of Aβ, a hallmark of AD. Studies demonstrate that β2-AR activation enhances autophagy by restoring lysosomal acidity and promoting the degradation of Aβ aggregates. Furthermore, β2-AR stimulation protects against Aβ-induced synaptotoxicity by supporting synaptic integrity and plasticity through downstream signaling pathways, such as the cAMP/PKA pathway. β1-ARs are also critical in AD, with their activation enhancing learning and memory processes via modulation of LTP and synaptic plasticity [55].

These neuroprotective effects extend beyond synaptic plasticity to include reductions in amyloid pathology and neuritic damage. Chronic stimulation of β2-ARs in preclinical models, such as the 5xFAD mouse model of AD, has resulted in lower amyloid plaque burdens and improved neuritic integrity, suggesting that microglial β2-AR signaling not only limits amyloid deposition but also preserves neurons [10]. The evidence supporting β2-ARs’ protective role is further strengthened by epidemiological studies. Retrospective analyses have suggested that individuals exposed to β2-AR agonists have a reduced risk of developing AD, with hazard ratios indicating a protective effect, while an increased risk was observed with patients receiving non selective β2 AR agonists. These findings align with preclinical data, supporting the idea that β2-AR activation can delay or prevent the onset of neurodegeneration [56]. Moreover, β2-ARs contribute to promoting neurogenesis and synaptic health, key factors in cognitive resilience. In APP/PS1 transgenic mice, β2-AR agonists such as clenbuterol enhanced neurogenesis, increased dendritic branching, and upregulated synaptic protein expression. These structural and functional improvements were accompanied by reductions in amyloid plaques, reduced APP phosphorylation, and amelioration of memory deficits, reinforcing the therapeutic promise of β2-AR-targeted treatments [57,58]. In addition to its effects on amyloid pathology, adrenergic signaling offers promise in targeting tau pathology. β-AR agonists such as salbutamol have been identified as potential inhibitors of tau aggregation. Salbutamol reduces tau filament formation and prevents the structural transition of tau into β-sheet-rich aggregates. This novel approach suggests that adrenergic signaling could address both major pathological pathways in AD, amyloid and tau, through distinct mechanisms [59].

All these positive effects of the β2 adrenergic receptor are corroborated by the fact that deletion of the β2-AR gene ameliorates pathological effects in senile PS1/APP mice, indicating that β2AR may represent a potential therapeutic target for preventing the progression of AD [60].

Adding to the therapeutic potential, non-pharmacological interventions such as aerobic exercise also leverage β2-AR activation to deliver benefits in AD. Aerobic exercise has been shown to reverse autophagy-lysosomal deficits and attenuate amyloid pathology through the upregulation of the AMPK-MTOR signaling pathway and VMA21 levels. These molecular changes enhance the clearance of Aβ and improve cognitive outcomes, illustrating the synergy between lifestyle interventions and β2-AR activation in managing AD [61].

### 1.6. Adrenergic Signaling, Beyond the Activity of Specific Receptor Subtypes

Adrenergic signaling, beyond the activity of specific receptor subtypes, has been increasingly associated with beneficial outcomes in neurodegenerative diseases, particularly AD. Activation of adrenergic pathways plays a critical role in preserving synaptic function and enhancing neuroprotective mechanisms, making it a promising area for therapeutic intervention. One key aspect of adrenergic signaling is its impact on synaptic plasticity, a process that is often impaired in AD. β-AR activation has been shown to enhance LTP, a mechanism underlying memory and learning. In the TgF344-AD rat model, the loss of LC-NA axons is compensated by increased β-AR, and this heightened β-AR function resulted in increased LTP magnitude and preserved learning and memory abilities. These findings suggest that β-AR activation could serve as a compensatory mechanism to counteract the synaptic dysfunction characteristic of AD [62]. Adding to this, vagus nerve stimulation, which indirectly activates β-ARs, has been found to modulate hippocampal synaptic transmission, offering potential cognitive benefits. This stimulation enhances noradrenaline release, leading to increased β-AR activation and improved synaptic transmission in the CA3 region of the hippocampus as the excitatory effect was diminished by the β-AR antagonist timolol. The ability to modulate hippocampal activity highlights the therapeutic potential of interventions that engage adrenergic pathways in addressing memory and learning deficits in AD [63]. Furthermore, β-adrenergic receptor activation plays a crucial role in mitigating Aβ-mediated synaptic impairment. Activation of both β1 and β2 adrenergic receptors by isoproterenol has been shown to prevent the inhibition of mossy fiber (axons) LTP caused by Aβ oligomers in mice. This neuroprotective effect underscores the potential of targeting specific β-AR subtypes to preserve synaptic function and combat Aβ-mediated damage in AD models [63].

Adrenergic signaling also extends its protective influence on microglial activity and amyloid clearance. Noradrenaline, acting through β1 and β2-ARs, has been demonstrated to prevent Aβ toxicity by stimulating NGF and BDNF in human neuronal cultures and primary rat hippocampal neurons [64,65]. Specific microglial β2-AR deletion worsened AD pathology in the 5xFAD mouse model, while chronic β2-AR stimulation resulted in attenuation of amyloid pathology and associated neuritic damage, suggesting microglial β2-AR might be used as a potential therapeutic target to modify AD pathology [10,66]. Additionally, NE can enhance amyloid clearance in murine microglia cell line N9 by involving upregulation of amyloid receptor expression and increased degradation of Aβ, driven by β2-AR activation. These findings highlight the dual role of adrenergic signaling in both protecting neurons from Aβ toxicity and reducing amyloid burden, addressing two critical aspects of AD pathology [64]. The anti-inflammatory properties of adrenergic signaling further strengthen its therapeutic potential. Studies on human THP-1 macrophages revealed that norepinephrine reduces Aβ-induced cytotoxicity and modulates cytokine secretion. This effect is mediated through β-AR activation, as the effects of NE were replicated by isoproterenol and blocked by propranolol. The effect is triggered by the cAMP/PKA signaling pathway and promotes CREB phosphorylation. By modulating immune responses, adrenergic signaling reduces inflammation and supports a more neuroprotective environment, further contributing to its benefits in AD [67].

Adrenergic signaling also plays a critical role in maintaining energy homeostasis in the brain, an often-overlooked aspect of neurodegenerative disease pathology. Noradrenaline, acting via β1-ARs, has been shown to regulate glycogen synthesis in astrocytes. This involves increased expression of protein targeting to glycogen (PTG) mRNA and enhanced glycogen synthesis, suggesting a role for adrenergic signaling in supporting metabolic demands and protecting neuronal health in energy-deficient states such as AD [68].

### 1.7. Negative Roles of β-Adrenergic Receptors in Alzheimer’s Disease and Other Neurodegenerative Disorders

#### 1.7.1. β1-Adrenergic Receptors

The β1-ARs, while often associated with therapeutic potential, have also been implicated in negative outcomes related to AD and other neurodegenerative disorders; however, with much less evidence stemming mainly from genetic studies. Polymorphisms in the β1-AR gene (*ADRB1*) and the G protein beta3 subunit (*GNB3*) gene have been identified as risk factors for AD. These genetic variations alter cell responsiveness to adrenergic stimulation, as evidenced by cAMP levels and mitogen-activated protein kinase (MAPK) activation and increased APP production in transfected human cell lines. These signaling alterations in specific polymorphisms may amplify pathological processes in AD, highlighting the genetic interplay between adrenergic receptor signaling and AD risk [69].

#### 1.7.2. β2-Adrenergic Receptors

The β2-ARs, while often linked to protective roles, have also been associated with several negative effects in AD. These receptors are deeply involved in synaptic function and neuronal signaling, but their dysregulation can contribute to the progression of AD through multiple mechanisms, including Aβ and tau pathology, synaptic dysfunction, and genetic predispositions. One key negative effect of β2-ARs in AD is their vulnerability to Aβ-induced internalization and degradation. Aβ peptides bind directly to β2-ARs, triggering their internalization and subsequent desensitization. This leads to impaired adrenergic and glutamatergic signaling, both of which are critical for synaptic function and plasticity. By attenuating responses to subsequent adrenergic stimulation, Aβ disrupts the homeostasis of neurotransmitter systems vital for learning and memory [70].

In addition to their interaction with Aβ, β2-ARs play a role in exacerbating tau pathology. In tauopathy models, deletion of the β2-AR gene has been shown to reduce tau pathology and improve motor deficits. Mechanistically, this is associated with reduced activity of tau kinases such as glycogen synthase kinase-3β (GSK3β) and cyclin-dependent kinase 5 (CDK5), which are known to phosphorylate tau and drive its pathological aggregation. These findings suggest that β2-AR signaling may contribute to tau hyperphosphorylation and accumulation, further linking β2-AR activity to neurodegeneration. This of course indicated that blockade of the receptors may be beneficial [71]. Altered β2-AR signaling also disrupts calcium homeostasis and APP processing, leading to additional synaptic dysfunction. Post-translational modifications of ryanodine receptors are regulated by β2-AR activity, resulting in abnormal calcium release that exacerbates βAPP processing. Blocking β2-AR signaling in cells has been shown to mitigate these effects, by reducing APP processing and Aβ production, indicating that β2-AR dysregulation may drive pathological APP cleavage and subsequent Aβ production, and β2 blockade could prove beneficial [72].

Further compounding its role in synaptic dysfunction, β2-AR activation contributes to Aβ-induced hyperactivity of AMPA receptors. This involves PKA-dependent phosphorylation of AMPA receptor subunits, which disrupts normal synaptic activity and exacerbates neuronal excitotoxicity. This effect is mediated by the binding of Aβ to β2-ARs [73]. The genetic component of β2-AR signaling dysfunction has also been implicated in AD. Polymorphisms in the β2-AR gene (Specifically Gly16Arg Gln27Glu) are associated with an increased risk of late-onset AD, particularly in conjunction with the APOE ε4 allele. These genetic interactions suggest that specific β2-AR variants may predispose individuals to AD by altering receptor function or adrenergic responsiveness [74].

The presence of agonistic autoantibodies targeting β2-ARs in AD patients further emphasizes their role in disease progression. These autoantibodies activate β2-ARs, mimicking adrenergic stimulation and potentially causing adrenergic overstimulation. Similar autoantibodies have been identified in other neurodegenerative disorders, such as glaucoma, suggesting a broader pathological mechanism linked to adrenergic overdrive. In addition, long-chain Aß-[Pyr]3-43, representing a major neurogenic plaque component, exerted an activation of the ß2-AR that could be blocked by antagonist ICI118.551 [75].

Another significant mechanism through which β2-ARs contribute to AD pathology is their interaction with the angiotensin II type 1 receptor in the production of Aβ, which seems to be more important than the β1-AR [76]. Activation of β2-ARs has been shown to enhance gamma-secretase activity, a key enzyme in Aβ production, thereby promoting amyloid plaque formation. This was also corroborated in vivo as chronic treatment with β2-AR agonist increased amyloid plaques in a mouse model of AD [77]. Stress-induced activation of β2-ARs further exacerbates this process, as evidenced by increased Aβ production in response to β2-AR agonists. Conversely, β2-AR antagonists have been shown to reduce Aβ production, highlighting a potential therapeutic approach for AD prevention [78,79]. However, β2-AR antagonism is not without risks. Inhibition of β2-ARs with ICI 118,551 has been linked to exacerbated cognitive deficits, tau hyperphosphorylation and amyloid pathology in 3XTg-AD mouse models. Specifically, β2-AR antagonism has been associated with increased Aβ levels and tau phosphorylation, suggesting that the timing and context of β2-AR modulation are critical in determining its therapeutic viability [80].

### 1.8. Negative Impacts of Adrenergic Signaling on Neurodegeneration

Neuroinflammation is another area where adrenergic signaling may have detrimental effects. While adrenergic pathways often exhibit anti-inflammatory properties, the chronic use of β-blockers in AD models has demonstrated pro-inflammatory effects in certain contexts. In APP mouse models, prolonged β-blocker administration was associated with increased markers of phagocytosis and heightened CNS inflammation. This was accompanied by impaired cognitive behavior, as evidenced by deficits in learning and memory tasks. These findings suggest that inappropriate modulation of adrenergic signaling can potentiate inflammatory processes, complicating efforts to target these pathways in neurodegenerative diseases [81].

The interaction between adrenergic and cholinergic systems also contributes to cognitive decline, particularly in aging and AD. Studies have shown that the combined blockade of muscarinic cholinergic and β-adrenergic receptors exacerbates learning and memory deficits in rodents. For instance, co-administration of scopolamine, a muscarinic cholinergic antagonist, and propranolol, a β-adrenergic antagonist, significantly impaired performance in learning tasks in rat models, while either scopolamine or propranolol alone had no effect. These findings underscore the interdependence of adrenergic and cholinergic systems in maintaining cognitive function and suggest that disruptions in either or both systems can synergistically exacerbate cognitive impairments [82].

## 2. Discussion

The role of adrenergic receptors in AD has emerged as a crucial yet complex area of investigation. The findings of this review highlight the duality of the adrenergic receptor role, showcasing both their therapeutic potential and their contributions to disease pathology. This nuanced understanding lays the groundwork for innovative therapeutic strategies, but also underscores the challenges inherent in targeting such a multifaceted system.

Adrenergic receptors, α1, α2, and β1 and β2 subtypes, are integral to various neural processes including synaptic plasticity, neurovascular regulation, and inflammation. Dysregulation of adrenergic signaling, often due to early degeneration of the locus coeruleus, is increasingly recognized as a driver of key AD pathologies such as Aβ accumulation, tau hyperphosphorylation, and chronic neuroinflammation. These findings align with the understanding that adrenergic receptors act at the intersection of cognitive, vascular, and immune pathways, making them critical modulators of both protective and pathological processes.

The evidence from the above in vitro and in vivo studies indicates that their role is naturally important; however, pharmacological modulation has not been always consistent as activation or blockade can have the same effect, making selective modulation difficult. Despite this, the evidence summarized in Table 1 and Table 2 indicates that the positive effects of adrenergic receptors are derived by pharmacological modulation, highlighting the possibility of being used in the clinic, and Table 3 shows the negative roles. Interestingly, from the in vivo and in vitro models, it seems that β receptor activation is protective (Table 2), while for α1- AR, the results are inconsistent, as studies show that both activation and blockade can have beneficial effects (Table 1). The protective role of β2 AR is more evident a notion that is also supported by others [11].

While trying to delineate whether activation or blockade is beneficial, evidence is hampered by the lack of clinical data. In fact, strong evidence from clinical studies for the benefit of adrenoreceptors in AD is lacking. A number of studies have explored the potential protective effects of antihypertensive drugs acting on β receptors in AD, but the results have been mostly inconclusive. For instance, a study analyzing 849,378 antihypertensive users found that β-adrenoceptor blockers conferred small protective effects against dementia compared to other antihypertensives [83]. Another study involving 69,081 individuals suggested that highly blood–brain barrier (BBB)-permeable β blockers were associated with a reduced risk of AD compared to low BBB permeability β blockers [84]. However, the protective effect may not be solely due to blood pressure reduction, and the role of β1 receptors remains speculative. The role of asthma drugs acting on β2 adrenergic receptors (β2AR) has been studied a lot less. A recent cohort study suggested that selective β2AR agonists are associated with a decreased risk of developing AD, while non-selective AR antagonists are associated with an increased risk [56]. With regard to the α-AR, in a double-blind placebo-controlled parallel-group study, Prazosin improved behavioral symptoms in patients with agitation and aggression in AD [85]. In a randomized double-blind placebo-controlled exploratory phase 2a trial with 100 subjects with AD and neuropsychiatric symptoms, ORM-12741 (a selective antagonist of alpha-2C adrenoceptors, dosed at 30–60 mg or 100–200 mg, b.i.d for 12 weeks) administered in addition to standard therapy with cholinesterase inhibitors showed a statistically significant effect in Quality of Episodic Memory [86].

Therefore, it still remains uncertain if and which adrenergic receptor modulation may be protective. However, we do believe that with the available dataset from patients and big data analysis using artificial intelligence and machine learning, it will be possible to make a comprehensive analysis using clinical data to determine if modulating the AR is beneficial and under what circumstances. It will indeed be important to make such studies as there are many AR drugs that can easily be repurposed for AD, other dementias or neurodegenerative diseases [87,88,89,90], while repurposing for other antihypertensive drugs is also possible [91,92] A number of properties makes this class of drugs an attractive novel therapy for AD: their proven safety and their multi target drug ligand property, which is thought to be a very promising method for targeting complex neurodegenerative diseases [93]. Given the failures observed with current clinical trials [94], even a small indication that these drugs may have efficacy will be a giant leap forward. Importantly, since some of these drugs cross the blood–brain barrier (BBB) fairly easily they can prove to be more efficacious than the current drugs in clinical trials [84,95,96].

While the review underscores the potential of adrenergic receptors as therapeutic targets, several challenges remain. One significant hurdle is the dual nature of receptor activity, where the same pathway can yield both beneficial and harmful outcomes depending on the context. This necessitates the development of highly selective modulators capable of fine-tuning receptor activity in a disease- and region-specific manner. Genetic studies further complicate this landscape, revealing polymorphisms in adrenergic receptor genes that interact with AD risk factors such as APOE ε4 [97]. These findings suggest that patient-specific factors [98,99,100], including genetic background and disease stage, must be considered when designing adrenergic-based interventions [69,74]. Additionally, the review highlights the need for more comprehensive models that accurately reflect the complexities of adrenergic dysfunction in AD. Current preclinical models often fail to capture the interplay between adrenergic, cholinergic, and serotonergic systems, which collectively shape the neurochemical environment in AD [101,102].

The findings of this review reaffirm the central role of adrenergic signaling in the pathophysiology of AD and other tauopathies. Adrenergic receptors offer a promising yet challenging target for therapeutic development, capable of modulating processes as diverse as neuroinflammation, synaptic plasticity, and amyloid clearance. Future research should focus on developing receptor subtype-specific drugs, exploring combination therapies that integrate adrenergic modulation with other neuroprotective strategies, and tailoring interventions to individual patient profiles. By addressing these challenges, adrenergic receptor modulation could pave the way for transformative therapies in the treatment of neurodegenerative diseases.

## 3. Conclusions

The review emphasizes the intricate role of adrenergic receptors in AD, highlighting both their therapeutic potential and their contribution to disease pathology. Adrenergic receptors are physiologically integral to neural processes such as synaptic plasticity, neurovascular regulation, and inflammation. Despite promising preclinical evidence suggesting that these receptors could be viable therapeutic targets, some preclinical studies point to their negative effects. What is more, strong clinical evidence is lacking, and the limited clinical studies thus far have yielded inconclusive results regarding the benefits of drugs acting on the β1 or β2 receptors. The dual nature of receptor activity, where both activation and blockade can potentially produce similar effects, presents a significant challenge in developing selective modulators. Additionally, genetic factors, patient-specific variables, the complexity of the disease itself, and the lack of good animal models, further complicate research in this area and therapeutic approaches. Future research should prioritize the development of subtype-specific drugs, combination therapies, and more controlled clinical trials with appropriate covariate selection, while also leveraging big data and advanced analytical techniques to refine strategies for adrenergic modulation in AD. This could potentially lead to innovative treatments not only for AD but also for other neurodegenerative diseases.

## Figures and Tables

**Table 1:** Protective roles of α adrenergic receptors by pharmacological modulation.

Receptor	Action	Modulator	Effects	References
**α1-AR**	Positive Allosteric Modulator	Cmpd-3	• Improves cognitive functions without affecting blood pressure in AD mouse models. • Enhances LTP, normalizes Aβ levels in AD models	[23]
	Agonists/Activators	Cirazoline, Avenanthramide-C	• Enhances cognitive functions, synaptic plasticity • Improves memory, reduces, neuroinflammation in AD models (effects blocked by prazosin -α1-AR inhibitor).	[24,25]
	Antagonists	Doxazosin, Prazosin, Terazosin	• Reduce Aβ generation and induces anti-inflammatory responses, prevent memory deficits • Improve cognitive functions reduce protein aggregates, and amyloid plaque burden. • Protect against amyloid-β toxicity, prevent GSK-3β activation and tau hyperphosphorylation.	[27,28,29,30]
**α2-AR**	Agonists	Brimonidine, Clonidine	• Offer neuroprotective effects by reducing apoptosis of retinal ganglion cells • Reduce Aβ and APP processing in vitro	[38]
	Antagonists	ORM-10921, Dexefaroxan, Mesedin, Idazoxan	• Improve cognitive functions, enhance neuron survival, reduces cholinergic degeneration, • Exhibits anti-amyloidogenic effects and ameliorate cognitive deficits.	[32,33,35,36,37]

**Table 2:** Protective roles of β adrenergic receptors by pharmacological modulation.

Receptor	Action	Modulator	Effects	References
**β1-AR**	Agonist	Xamoterol, STD-101-D1	• Neuroprotection, reduction inflammation, and synaptic dysfunction. • Enhance cognitive functions, memory, and social learning • Reduce Aβ and tau pathology	[48,49,51]
**β2-AR**	Agonist	Isoproterenol (via β2), Clenbuterol, Salbutamol	• Restore lysosomal function and autophagy, promote degradation of Aβ. • Enhance neurogenesis, synaptic health, and cognitive function • Reduce amyloid plaques, mitigates Aβ-induced inflammation and synaptotoxicity, decrease neuroinflammatory responses.	[52,53,54,55,56,57,58,59]
**Non-pharmacological**	Leverage β2 AR Activation	Aerobic exercise	• Reverses autophagy-lysosomal deficits; attenuates amyloid pathology, improves cognitive outcomes	[61]

**Table 3:** Negative roles of α and β adrenergic receptors by pharmacological and non-modulation.

Receptor	Modulator/Action	Effects/Implications	References
**α1-AR**	Agonistic autoantibodies (agAABs)–Prolonged Activation	• Non-physiological calcium rise, reduced blood flow, neurodegeneration, amyloid plaques, vascular lesions	[21,39,41]
	Aβ peptides–Activation	• Vascular dysfunction, intracellular calcium release in smooth muscle cells	[43]
	α1-AR–Upregulation in AD	• Aggressive behavior in AD patients	[44]
**α2-AR**	α2-AR–Reduced density in AD brains	• Loss of presynaptic receptors, cognitive & behavioral impairments	[45,46]
**β1-AR**	β1-AR gene (*ADRB1*) and G protein beta3 subunit (*GNB3*) gene–Polymorphisms	• Altered adrenergic response, amplifies AD pathology	[69]
**β2-AR**	β2-AR Activation–Aβ peptides or other	• Impaired neurotransmitter signaling, disrupting learning and memory • Enhances gamma-secretase, promoting amyloid plaques • Contributes to tau hyperphosphorylation and neurodegeneration	[70,71,77]
	β2-AR–Altered signalling	• Disrupts calcium homeostasis, APP processing, synaptic dysfunction	[72]
	Gly16Arg, Gln27Glu–Polymorphisms	• Increased late-onset AD risk (APOE ε4 synergy)	[74]
	Inhibition–ICI 118,551	• Cognitive deficits, tau hyperphosphorylation, amyloid pathology	[80]

## Data Availability

No new data were created in this study. Data sharing is not applicable to this article.
